# Small Molecule-Induced Complement Factor D (Adipsin) Promotes Lipid Accumulation and Adipocyte Differentiation

**DOI:** 10.1371/journal.pone.0162228

**Published:** 2016-09-09

**Authors:** No-Joon Song, Suji Kim, Byung-Hyun Jang, Seo-Hyuk Chang, Ui Jeong Yun, Ki-Moon Park, Hironori Waki, Dean Y. Li, Peter Tontonoz, Kye Won Park

**Affiliations:** 1 Department of Food Science and Biotechnology, Sungkyunkwan University, Suwon 16419, Korea; 2 Department of Diabetes and Metabolic Diseases, Graduate School of Medicine, University of Tokyo, Tokyo 113–8655, Japan; 3 Department of Medicine, Program in Molecular Medicine, University of Utah, 15 North 2030 East, Salt Lake City, UT, 84112, United States of America; 4 Howard Hughes Medical Institute and Department of Pathology and Laboratory Medicine, University of California Los Angeles, Los Angeles, CA, 90095, United States of America; Tohoku University, JAPAN

## Abstract

Adipocytes are differentiated by various transcriptional cascades integrated on the master regulator, Pparγ. To discover new genes involved in adipocyte differentiation, preadipocytes were treated with three newly identified pro-adipogenic small molecules and GW7845 (a Pparγ agonist) for 24 hours and transcriptional profiling was analyzed. Four genes, Peroxisome proliferator-activated receptor γ (Pparγ), human complement factor D homolog (*Cfd*), Chemokine (C-C motif) ligand 9 *(Ccl9)*, and GIPC PDZ Domain Containing Family Member 2 *(Gipc*2) were induced by at least two different small molecules but not by GW7845. *Cfd* and *Ccl9* expressions were specific to adipocytes and they were altered in obese mice. Small hairpin RNA (shRNA) mediated knockdown of Cfd in preadipocytes inhibited lipid accumulation and expression of adipocyte markers during adipocyte differentiation. Overexpression of Cfd promoted adipocyte differentiation, increased C3a production, and led to induction of C3a receptor (C3aR) target gene expression. Similarly, treatments with C3a or C3aR agonist (C4494) also promoted adipogenesis. C3aR knockdown suppressed adipogenesis and impaired the pro-adipogenic effects of Cfd, further suggesting the necessity for C3aR signaling in Cfd-mediated pro-adipogenic axis. Together, these data show the action of Cfd in adipogenesis and underscore the application of small molecules to identify genes in adipocytes.

## Introduction

Adipocytes, energy reservoirs in vertebrate organisms, have the machinery required for lipolysis, glucose uptake, and triglyceride synthesis [1,2]. Adipocytes are also an endocrine organ that secretes fatty acids and adipokines to regulate systemic metabolism upon various stimuli including hormones, feeding, and exercise[3]. Numerous studies have identified regulators including Pparγ in adipocytes. Pparγ is necessary and sufficient for adipogenesis [4]. Pparγ upstream regulators including CCAAT/enhancer binding protein (C/ebp), Early B cell factor, Interferon regulatory factor, Kruppel -like factor, early growth response, Inhibitor of DNA binding, Zinc finger proteins, and its downstream target genes have shown for their roles in lipid synthesis and adipocyte differentiation [5,6,7]. As Pparγ would be regulated by various integrated pathways, identification of a broader window of Pparγ upstream and its target genes linked to adipogenesis would provide further understanding of Pparγ and adipocyte biology.

Complement generated mainly by hepatocytes functions within innate immune defense [8,9]. A recent study showed that the adipose tissue produces complement component 3 (C3), complement factor B (Cfb), and complement factor D (Cfd, also called adipsin), factors required for the production of C3a [10]. Most of the other complement factors are also secreted by the adipose tissue, suggesting non-immunogenic roles of complement in adipose tissues. Indeed, C3a is recognized by C3aR, which triggers triglyceride synthesis and its levels are a risk factor for developing diabetes [11,12,13,14]. Acylation stimulating protein (ASP, C3adesArg), a C3 cleavage product by exopeptidase activity, binds to C5L2 and also promotes triglyceride synthesis [15]. Thus, complement factors including Cfd in immune cells and adipocytes may be the mediators for interweaving energy metabolism and immune responses.

We have previously identified various pro-adipogenic small molecules. Harmine was identified as a Pparγ inducer that promoted adipocyte differentiation of preadipocytes and increased insulin sensitivity without any signs of deleterious effects associated with Pparγ activation in diabetic mice [16]. Similarly, another small molecule phenamil has been shown to induce tribbles-like 3, resulting in stimulation of Bone morphogenetic protein signaling and Pparγ induction [17,18]. These studies indicate that small molecule Pparγ inducers may be a useful strategy to delay the onset of obesity- related metabolic diseases.

In this study, a previously developed cell-based high throughput screening assay was performed and it led to the identification of new pro-adipogenic small molecules. We chose and utilized three small molecules as tools to identify adipogenic genes. Transcriptional profiling assays of these selected small molecules revealed potential new adipogenic regulators including *Pparγ*, *Cfd*, *Ccl9*, and *Gipc*2. Among these, Cfd was originally described as a adipocyte-specific gene, but its role in adipocyte differentiation remains unknown [19]. Hence, we further focused on Cfd to investigate its role in adipogenesis. We showed that Cfd induced by adipogenic small molecules exerts pro-adipogenic effects and its actions can be mediated by activating C3a-C3aR pathways. These studies provide evidence for previously unknown roles of Cfd in adipocyte differentiation and further highlight the utility of pro-adipogenic small molecules in identification of the genes involved in adipocyte biology.

## Materials and Methods

### Identification of pro-adipogenic small molecules

Cell-based screening was performed as previously described [16]. Briefly, F442-Luc20 cells were plated and the chemical libraries were transferred using a pin tool. Five days later, cells were lysed and luciferase activity was determined. Small molecule libraries (Combichem Library) and screening were performed in UCLA MSSR core facility. A cell-based high throughput screening assay yielded a number of pro-adipogenic compounds [16]. Thirty-five compounds (top 0.02%) were selected from 160,000 small molecule libraries (Figure A in S1 File). Based on the strong effects of small molecules on Pparγ expression (> 2 folds) in 24 hours, 15 compounds were further selected Figure A in S1 File). These small molecules were then assessed for the stimulatory effects on adipocyte differentiation in 3T3-F442A cells and 3T3-L1 cells (Figure A in S1 File). Ppar activation assays were also performed to exclude small molecules acting as Pparγ agonists (Figure A in S1 File). GW7845 and phenamil were used as the control for Pparγ agonists and Pparγ inducers, respectively. These assays led to the identification of three new small molecules named PT7 (3-chloro-N-3- pyridinyl-1-benzothiophene-2- carboxamide), PT24 (N-1,3- benzodioxol- 5-yl-2- (2-thienyl) -4-quinolinecarboxamide), and PT26 (3- [3- (4-fluorophenyl) acryloyl]- 4,6-dimethyl-2 (1H)-pyridinone) (Figures A and B in S1 File).

### Cell culture

C3H10T1/2, 3T3-F442A, and 3T3-L1 cells were maintained as previously described [20,21]. In brief, C3H10T1/2 cells were grown in Dulbecco’s modified Eagle’s medium (DMEM) (Hyclone, Logan, UT) supplemented with 10% Fetal bovine serum (FBS, Hyclone) and antibiotics. 3T3-L1 preadipocytes were grown in DMEM containing 10% calf serum (CS) (Hyclone) and antibiotics. Confluent cells were differentiated into adipocytes by changing media containing DMEM, 10% FBS, antibiotics, 1 μM dexamethasone (Sigma, St. Louis, MO, USA), 0.5 mM isobutyl-1-methylxanthine (Sigma), and 5 μg/ml insulin for 2 days. The cells were refreshed with DMEM supplemented with 10% FBS and 5 g/ml insulin every 2 days. 3T3-F442A preadipocytes were induced into adipocytes by treating with 5 μg/ml insulin (Sigma) in DMEM containing 10% FBS. Troglitazone (1 μM, Sigma) was additionally added to induce adipocyte differentiation of C3H10T1/2 cells. C3 and C3a were purchased from CompTech (Tyler, TX, USA) and C3a agonist C4494 was from Sigma. C3a contents were measured by ELISA as per the manufacturer’s instructions (MyBioSource, San Diego, CA, USA). Differentiated adipocytes were fixed with 4% paraformaldehyde and stained with 0.5% Oil Red O (Sigma). Oil Red O stained cells from at least two independent experiments were extracted with isopropanol and measured with a spectrophotometer at 520 nm.

### Gene expression analysis

Microarray was performed as described previously [17,18]. Total RNAs from 24 hour pro-adipogenic small molecule treated F442A cells were extracted using TRizol reagent (Invitrogen, Carlsbad, CA) and further purified using RNAeasy columns (QIAGEN, Chatsworth,CA). cDNA preparation and hybridization to Mouse-6 expression Beadchip were performed by the UCLA core facility.

Total RNA was extracted by TRIzol (Invitrogen) and was used to synthesize complementary DNA (cDNA) using ReverTra Ace^®^ qPCR RT Master Mix (TOYOBO, Osaka, Japan). The synthesized cDNA was subjected to 40 PCR-amplification cycles using THUNDERBIRD^®^ SYBR^®^ qPCR Mix (TOYOBO) and primers in a Thermal Cycler Dice (Takara, Shiga, Japan). All real-time PCR reactions were performed at least twice. The following primers were used for amplification: *Cfd*: *Cfd* F, 5’—ctgggagcggctgtatgt-3’ and Cfd R, 5’- cacggaagccatgtaggg-3’; Ccl9: Ccl9 F, 5’- tgggcccagatcacacat-3’ and Ccl9 R, 5’- cccatgtgaaacatttcaatttc-3’; Gipc2, Gipc2 F, 5’- tggggattcgagatattgactt-3’ and Gipc2 R, 5’- ctcatctgggttgctcttgtc-3’. Other primers (Integrated DNA Technologies, San Diego, CA) were described previously [21,22]. The expression of genes was normalized to 36B4 and the relative expression level was calculated using 2^-ΔCT^.

Male 7 week olds C57BL/6 mice were purchased from Central Lab Animal Inc. (Seoul, Korea). After 1 week of adaption, the mice were divided into two groups. The mice in one group were fed a normal diet (ND, n = 8) and the other group (n = 8 per group) were fed a high-fat diet containing 60% calories from fats (HFD; D12492, Research Diets Inc., New Brunswick, NJ, USA) and individually housed in a temperature-controlled room with a 12-hour light/dark cycle for 8 weeks. The mice were euthanized and adipose tissues were dissected to isolate RNAs. Homogenized adipose tissues with TRIzol were centrifuged to remove lipid layer. The isolated total RNAs were reversely transcribed and the synthesized complementary DNA was used for realtime PCR analysis. Mice were euthanized by carbon dioxide inhalation and immediately exsanguinated by cardiac puncture. Animal studies were carried out in accordance with the recommendations in the Guide for the Care and Use of Laboratory Animals of the National Institutes of Health. The protocol was approved by the Committee on the Ethics of Animal Experiments of the Sungkyunkwan University (Permit Number: SKKUIACUC-20150037).

### Knockdown studies

The scrambled control and Cfd-specific shRNA sequences were synthesized by Genolution Pharmaceuticals, Inc. (Seoul, Korea). To generate stable cells, shRNAs were cloned into pRetroSuper and Phoenix E packaging system was used as described [23]. The sense sequences of two independent *Cfd*-specific and C3aR-specific shRNAs were as follows: *Cfd* sh #1: 5′-GCUAAUGGUACCUCUUUCAUU-3′, *Cfd* sh #2: 5′- CAUUGAUGACACAUUCUCUUU -3′. For *C3aR silencing*, siRNAs were transfected for 12 hours using Lipofectamine RNAiMAX (Invitrogen). A concentration of 30 nM siRNAs was transfected into 80% confluent 3T3-L1 cells. The cells were replenished with fresh medium after 12 hours and 48 hours later the cells were differentiated into adipocytes. Transfection was repeated at least three times. The sense sequence of the control, nonspecific scrambled RNA was 5′CCUCGUGCCGUUC CAUCAGGUAGUU -3′. The sense sequences of two independent *C3aR*-specific siRNAs (shRNAs) were as follows: *C3aR* si #1: 5’- GCUGCUCUUAUUGUUCUGAAUUU-3’, *C3aR* si #2: 5’- CCAGCCUCUUCUUUAUCAUUAUU-3’.

### Overexpression studies

HEK293T cells were transfected for 48 hours with pBabe-puro or pBabe-puro-Cfd and retroviral packaging vectors [21]. Polybrene (8 ng/ml, Sigma) was further added and viral supernatants were harvested and filtered. C3H10T1/2 or 3T3-L1 cells were exposed to viruses for 24 hours and then stable cells were selected by puromycin treatment for 2 weeks and differentiated into adipocytes.

### Statistical analysis

Data are presented as the mean ± SEM. Statistical significance differences were determined using one- way ANOVA and the Student-Newman-Keuls test. Differences in gene expression and lipid accumulation in two groups were analyzed using a two-tailed unpaired Student’s *t*-test. Statistical significance was defined as *P* < 0.05.

## Results

### Complement factor D (Cfd) is induced by pro-adipogenic small molecules

**Fig 1:**
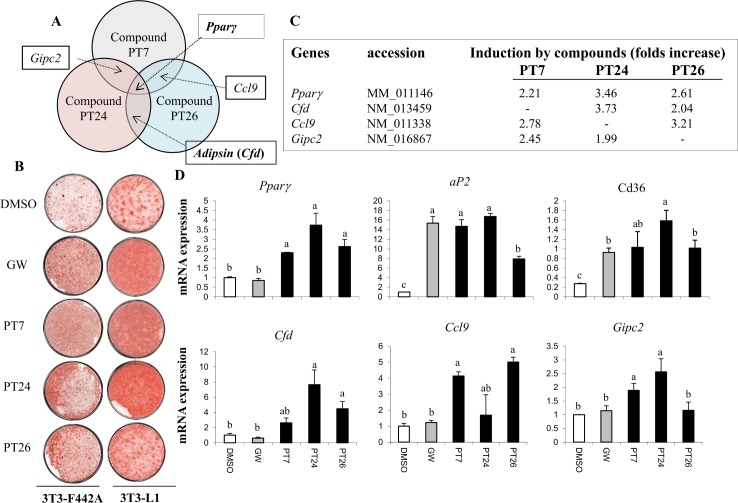
Induction of *Cfd* by adipogenic small molecules. (**A**) A diagram showing the overlap genes induced by pro-adipogenic small molecules. 3T3-F442A preadipocytes were treated with GW7845 or compounds and the induced genes (>2.0 folds) were identified by microarray analysis. Pparγ agonist (GW7845)-induced genes (>2.0 folds) were excluded from the candidates. Pparγ is the overlapping gene induced by three adipogenic compounds. *Gipc2*, *Ccl9*, and *Cfd* were induced by at least two pro-adipogenic compounds. (**B**) Pro-adipogenic effects of the selected compounds were verified in 3T3-F442A and 3T3-L1 cells. The confluent 3T3-F442A preadipocytes were stimulated with adipogenic medium containing DMEM, 10% FBS, insulin, and the selected compounds for 6 days. Lipid accumulation was assessed with Oil red O staining. The 3T3-L1 preadipocytes were induced into adipocytes in adipogenic medium containing DMEM, 10% FBS, dexamethasone, IBMX, insulin, and the selected compounds. Then, the 3T3-L1 cells were refreshed with media supplemented with insulin and compounds every 2 days until day 6. (**C**) Fold induction of pro-adipogenic small molecule-induced genes compared to DMSO treated cells is shown. (**D**) Expression of *Cfd*, Ccl9, and *Gipc2* induced by the selected compounds was verified in 3T3-F442A cells. 3T3-F442A preadipocytes were treated with GW7845 and the selected compounds for 24 hours. Induction of adipocyte markers was assessed by real time PCR. Data are presented as means +/- s.d. and are representative of three independent experiments. Statistical significance differences were determined using one- way ANOVA and the Student-Newman-Keuls test. Different letter at each samples are significant (*p* <0.05)

Three new adipogenic small molecules were identified by a previously developed cell-based high throughput screening assay [16]. To investigate the transcriptional profiling, 3T3-F442A cells were treated with selected small molecules or a Pparγ agonist GW7845 for 24 hours and microarray analysis was performed (Figure B in S1 File, Fig 1A and 1B). Direct Pparγ target genes were excluded by comparing with the list of genes induced by GW7845. Numerous genes specific to a single compound and overlapped genes induced by at least two compounds were identified (Fig 1C). We hypothesized that specific genes would be more related to the unique mechanism of each compound, whereas genes commonly induced by three small molecules could be convergent genes acting on promoting Pparγ expression and adipocyte differentiation. As we were interested in identifying new players in adipocyte differentiation and Pparγ inducers, we focused on the overlapped genes. Only four genes were regulated by at least two different small molecules. Pparγ was induced by all three compounds but not by GW7845 treatments for 24 hours, further verifying the approaches. Other candidate genes included *Ccl9* induced by PT7 and PT26, *Gipc*2 induced by PT7 and PT24, and *Cfd* induced by PT24 and PT26 (Fig 1C and 1D).

**Fig 2:**
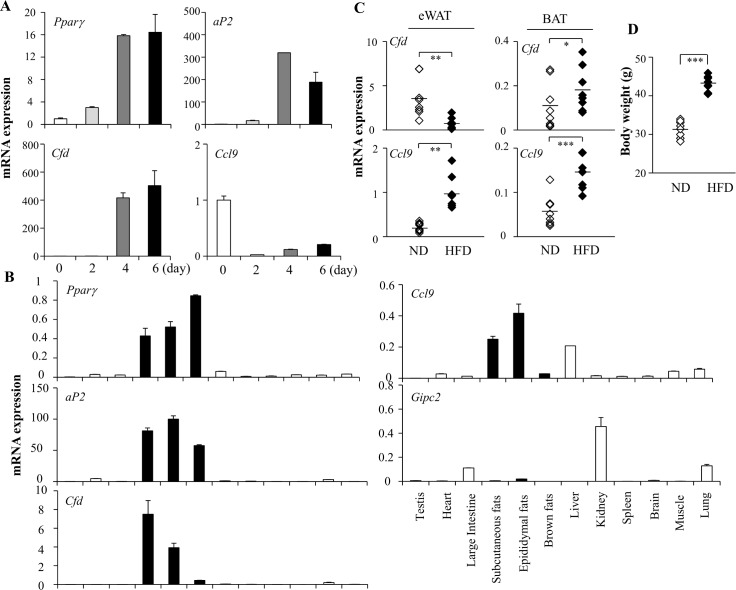
Expression profiles of *Ccl9* and *Cfd* during adipocyte differentiation and in adipose tissues. (**A**) Induction of *Pparγ*, *aP2*, *Cfd*, and *Ccl9* by adipogenic cocktail in 3T3-L1 cells. mRNA levels were assessed by real-time PCR at various time points over 6 days after treatment with a defined adipogenic cocktail including dexamethasone, isobutylmethylxanthine, and insulin (DMI). (**B**) Tissue distribution of *Cfd*, *Ccl9*, *Gipc2*, *Pparγ*, and aP2 in mice. mRNA expression was measured by real-time PCR. Data shown represent the mean ± s.e.m (n = 3). (**C**) *Ccl9 and Cfd* mRNA expression in epididymal fat tissues (eWAT) and interscapular brown adipose tissues (BAT) was measured in normal diet-fed (ND) mice and high-fat diet-fed (HFD) obese mice. (D) Body weights of normal diet and HFD fed mice. Data shown represent the mean +/- s.e.m (n = 8 per group). Statistical significance was determined relative to a control by the Student’s *t*-test (* *P* <0.05; ** *P* <0.005; *** *P* <0.0005).

We next investigated the expression profiles of these genes during adipocyte differentiation of 3T3-L1 cells and in mice. Similar to the *aP2* and *Pparγ* expression profiles, *Cfd* expression was increased during adipocyte differentiation. *Ccl9* expression was promptly decreased at day 2 and then moderately increased during adipogenic processes (Fig 2A). Unlike expression patterns in 3T3-F442A cells, *Gipc2* expression was not detectable in 3T3-L1 cells. In tissues, *Cfd* expression was adipose tissue selective with highest expression in epididymal fat (eWAT). *Ccl9* was also highly expressed in adipose tissues. *Gipc2* expression was high in kidney, lung, and intestine, but it was almost excluded from fat tissues (Fig 2B). The expression profiles of *Ccl9* and *Cfd* in adipocytes are consistent with previous data [24,25]. *Ccl9* expression was increased in eWAT and brown adipose tissues (BAT) from HFD fed obese mice compared to control lean mice. *Cfd* expression was defective in obese mice (Fig 2C and 2D). We focused on Cfd at this stage to further investigate its roles in adipocytes.

### Complement factor D (Cfd) promotes adipocyte differentiation

**Fig 3:**
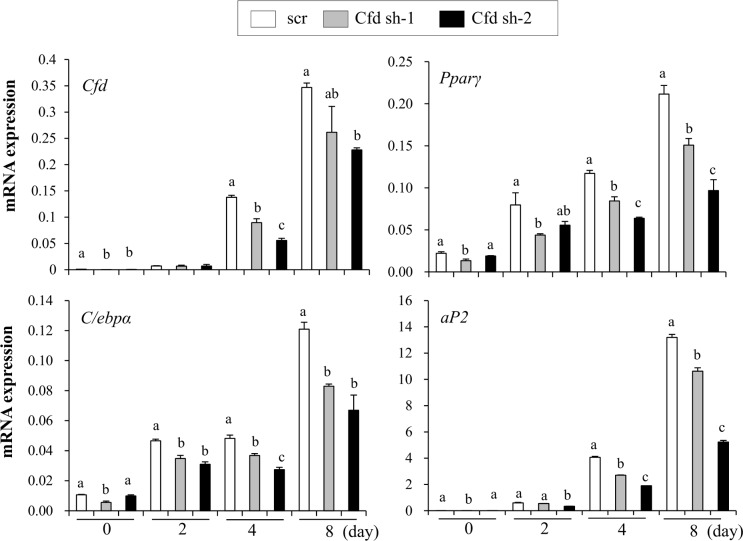
Silencing of Cfd suppresses adipocyte differentiation. Stable C3H10T1/2 cells expressing two independent shRNAs against *Cfd* or scrambled shRNA were generated. Stable cells were differentiated into adipocytes for 8 days and expression of *Cfd*, *Pparγ*, *C/ebpα*, and *aP2* was measured by real time PCR analysis. Data are presented as means +/- s.e.m. and are representative of two independent experiments. Each independent experiment was carried out in triplicate. Statistical significance differences were determined using one- way ANOVA and the Student-Newman-Keuls test. Different letter at each samples are significant (*P* <0.05)

**Fig 4:**
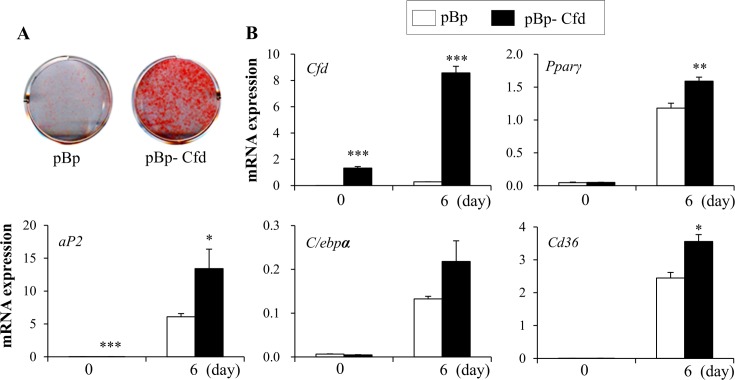
Stable overexpression of Cfd promotes lipid accumulation and adipocyte differentiation. 3T3-L1 cells were infected with pBabe-puro empty vector (pBp) or pBabe-Cfd gene harboring retrovirus (pBp-Cfd) and stable pools selected using puromycin (2 ug/ml) for 2 weeks. The stable 3T3-L1 preadipocytes were induced into adipocytes in adipogenic medium containing DMEM, 10% FBS, dexamethasone, IBMX, insulin, and the selected compounds. Then, the 3T3-L1 cells were refreshed with media supplemented with insulin and compounds every 2 days. (**A**) Stable cells were differentiated into adipocytes for 6 days and lipid accumulation was assessed by Oil red O staining. (**B**) Expression of *Cfd* and adipocyte markers was measured on day 0 and day 6 of differentiation by real time PCR analysis. Data are presented as means +/- s.e.m. and are representative of three independent experiments. Each independent experiment was carried out in triplicate. Statistical significance was determined relative to a control by the Student’s *t*-test (* *P* <0.05; ** *P* <0.005; *** *P* <0.0005).

To test whether Cfd can stimulate lipid accumulation, we stably expressed retrovirus harboring shRNAs targeting Cfd in C3H10T1/2 cells and induced to differentiate into adipocytes. As expected, *Cfd* expression was lower in Cfd-shRNA cells compared to control cells (Fig 3). Two independent shRNA-expressing cells showed suppressed expression of *Pparγ* and its target genes during adipocyte differentiation compared to control (scr) cells (Fig 3). To further confirm this, we stably overexpressed *Cfd* in 3T3-L1 cells using pBabe retroviral system. Cfd stably overexpressing retrovirus infected cells (pBp-Cfd) compared to empty vector expressing virus infected cells (pBp) showed increased lipid accumulation (Fig 4A). In addition, adipocyte markers were increased in Cfd overexpressing cells (Fig 4B). Consistent effects by Cfd overexpression in C3H10T1/2 cells were also observed (Figure C in S1 File). Taken together, loss and gain of function studies can assign the role of Cfd during adipocyte differentiation.

### Complement factor D induces C3aR activation

**Fig 5:**
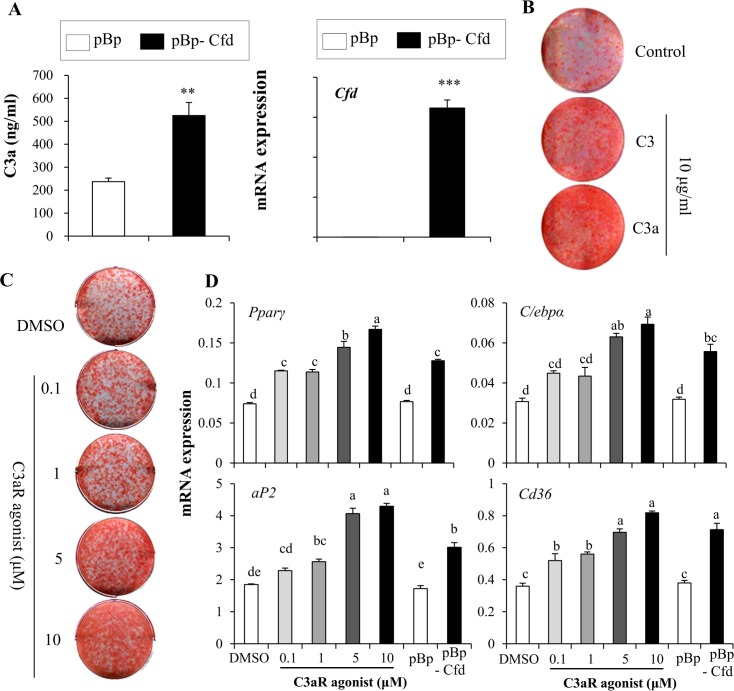
*Cfd* promotes adipocyte differentiation through C3a-C3aR activation. (**A**) Increased C3a production was observed in Cfd stably overexpressing 3T3-L1 cells. Media was collected from control or Cfd overexpressing cells and C3a content was measured by ELISA as described in methods. (**B**) Treatment with C3 or C3a increased lipid accumulation. (**C**) C4494, a known C3a agonist, stimulated lipid accumulation in a dose-dependent manner in 3T3- L1 cells. (**D**) Expression of Pparγ, *C/ebpα*, *aP2*, and *Cd36* was also increased by C4494. 3T3-L1 cells stably expressing pBabe-puro empty vector (pBp) or pBabe-Cfd gene harboring retrovirus (pBp-Cfd) were used as controls. Data are presented as means +/- s.d. and are representative of three independent experiments. Statistical significance differences were determined using one- way ANOVA and the Student-Newman-Keuls test. Different letter at each samples are significant (*P* <0.05)

Cfd is a serine protease secreted by adipocytes and it cleaves factor B, resulting in increased C3a and C3b production [9]. C3a has also been shown to promote triglyceride synthesis in adipose tissue [26]. Based on these, we reasoned that induced levels of C3a by Cfd can be responsible for increases in lipid accumulation and adipocyte differentiation. To test this possibility, we measured C3a production in media from control (pBp) and Cfd overexpressing 3T3-L1 preadipocytes (pBp-Cfd). C3a production was three times higher in pBp-Cfd cells compared to control pBp cells (Fig 5A). These data show the possibility that increased C3a production, at least in part, can mediate the pro-adipogenic effects in Cfd expressing cells.

### C3aR signaling stimulates adipocyte differentiation

To further directly investigate the role of C3a in adipocytes, we treated 3T3-L1 cells with C3 or C3a and induced to differentiate into adipocytes. Similar to the effects by Cfd, treatment with either C3 or C3a stimulated lipid accumulation (Fig 5B). Furthermore, a C3aR (C3a receptor) agonist C4494 also increased lipid accumulation and expression of adipocyte markers comparable to the levels in Cfd overexpressing 3T3-L1 cells (Fig 5C and 5D).

**Fig 6:**
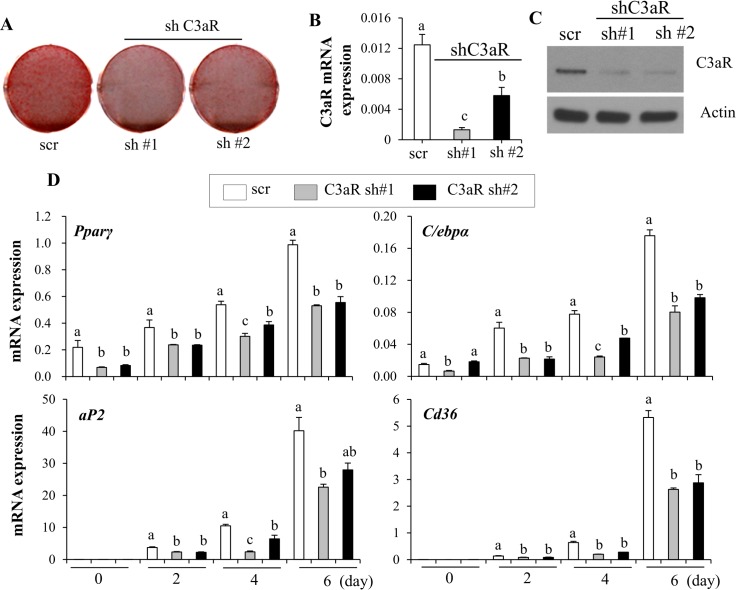
C3aR signaling is essential for adipocyte differentiation. (**A**) Knockdown of C3aR inhibits adipocyte differentiation. C3H10T1/2 cells were infected with virus harboring two independent shRNAs against C3aR or scrambled shRNA (scr) and stable cells were selected with puromycin (2 μg/ml) for 2 weeks. Stable C3H10T1/2 cells were differentiated into adipocytes and lipid accumulation was assessed by Oil red O staining. (**B**) Two independent shRNAs effectively reduce C3aR mRNA expression. (**C**) C3aR protein expression in control and C3aR knockdown cells was measured by western blotting. (**D**) Expression of *Pparγ*, *C/ebpα*, *aP2*, and *Cd36* in C3aR-deficient C3H10T1/2 cells was measured by real time PCR analysis. Data are presented as means +/- s.d. and are representative of two independent experiments. Each independent experiment was carried out in triplicate. Statistical significance differences were determined using one- way ANOVA and the Student-Newman-Keuls test. Different letter at each samples are significant (*P* <0.05)

Since C3a can exert its effects through interaction with the receptor C3aR, we investigated the role of C3aR in adipocytes. To test the actions of C3aR in adipogenesis, we silenced C3aR using two independent shRNAs and assessed the effects on adipocyte differentiation. In C3aR deficient C3H10T1/2 cells, lipid accumulation was decreased compared to scrambled shRNA transfected control (scr) cells (Fig 6A). Expression of C3aR and adipocyte markers was consistently decreased in the C3aR deficient cells (Fig 6B–6D). Furthermore, C3aR expression was also necessary for adipocyte differentiation in 3T3-L1 cells (Figure D in S1 File), exhibiting that the C3aR signaling promotes lipid accumulation and adipocyte differentiation.

### C3aR is required for Cfd action in adipocytes

**Fig 7:**
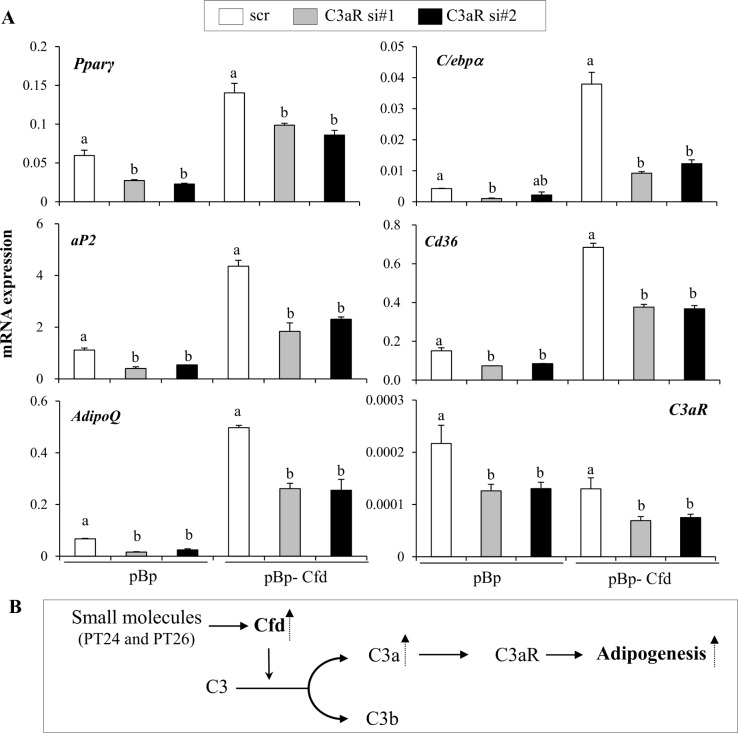
C3aR signaling is required for adipogenic potential of Cfd overexpressing cells. (**A**) The increased adipogenic potential of Cfd expressing cells is blocked by silencing C3aR expression. Control empty vector (pBp) or Cfd stably overexpressing 3T3-L1 cells (pBp- Cfd) were transiently transfected with siRNAs targeting C3aR and differentiated into adipocytes for 6 days. Expression of adipocyte markers was assessed by real time PCR. Data are presented as means +/- s.d. and are representative of two independent experiments. Each independent experiment was carried out in triplicate. Statistical significance differences were determined using one- way ANOVA and the Student-Newman-Keuls test. Different letter at each samples are significant (*P* <0.05). (**B**) A schematic model of small molecules-induced Cfd in C3a-C3aR mediated adipogenesis.

To test whether Cfd can promote adipocyte differentiation by acting on C3aR signaling, we overexpressed Cfd and concomitantly silenced C3aR expression in 3T3 preadipocytes. Interestingly, increased expression of *Pparγ* in Cfd overexpressing cells (pBp-Cfd) was significantly impaired in the C3aR-deleted cells (C3aR si#1 and #2). Induction of Pparγ target genes including *C/ebpα*, *aP2*, *Cd36*, and *adiponectin* (*adipoQ*) by Cfd overexpression was also blunted in C3aR-silenced cells (Fig 7A). These data suggest the requirement of C3aR signaling, at least in part, for Cfd-mediated pro-adipogenic effects.

Based on the possibility that C3aR signaling is crucial for the effects of Cfd, we treated pBp-Cfd and pBp cells with C3aR agonist (C4494) and compared the adipogenic potentials. C3aR-activated cells showed higher levels of lipid accumulation compared to DMSO-treated cells. C3aR activation in pBp-Cfd cells, however, failed to further induce lipid accumulation. Similarly, the expression levels of adipocyte markers (*aP2*, *C/ebpα*, and *Cd36*) were higher in C3aR activated pBp cells, but C3aR activation in pBp-Cfd cells did not further produce synergistic induction of adipocyte genes (Figure E in S1 File). These data show the functional importance of C3aR signaling in Cfd-mediated pro-adipogenic effects (Fig 7B).

## Discussion

Previously, bioactive small molecules were utilized to drag out critical players in cell biology [27]. MyoD in myogenesis and Pparγ in glucose metabolism were previously revealed by investigating the molecular actions of small molecules [28,29,30]. Similarly, pro-adipogenic small molecules can also be used as a tool to discover new players in biological processes. Toward this end, we utilized a previously developed cell-based high throughput screening assay to identify pro-adipogenic small molecules. Top 35 small molecules (top 0.02%) were initially selected to test their effects on promoting adipogenesis and Ppar*γ* expression in 3T3-F442A cells. From these candidates, we further selected three most potent pro-adipogenic compounds (named PT7, PT24, and PT26). Molecular signatures of these selected Pparγ inducers were analyzed and these led to the identification of four genes (*Pparγ*, *Cfd*, *Ccl9*, and *Gipc2*). Although we investigated the previously unknown adipogenic roles of Cfd in the current study, it is also possible that other genes such as Ccl9 and Gipc2 can be potential candidates for Pparγ induction as shown in impaired adipocyte differentiation in Ccl9- silenced cells (Figure F in S1 File). As Pparγ upstream factors such as CDK5 are considered as new molecular targets for insulin resistance and other metabolic diseases, mechanism studies of these compounds may provide new molecular targets for obesity and obesity-related metabolic diseases [31,32].

Cfd is a differentiation dependent serine protease secreted by adipocytes and its expression is deficient in obese and diabetic disease mice models [33,34,35]. Furthermore, recent studies show that Cfd stimulates insulin secretion and maintains β cell function through C3a-C3aR signaling [36]. Cfd is also involved in triacylglycerol synthesis in human adipocytes and C3a levels are a risk factor for developing diabetes [11,12,13,14]. An association between familial C3 deficiency and partial lipodystrophy [37] also suggests the roles of complement system in pathological conditions associated with obesity and its related metabolic diseases [33,34]. In this study, we showed that Cfd promoted adipocyte differentiation and its effects can be mediated by stimulating C3aR signaling pathways. Therefore, it is possible that Cfd may function in both adipocytes and pancreatic β cells to serve as a critical player in obesity and diabetes [36]. Accordingly, it would be worth to further investigate the actions of Cfd in obesity and metabolic diseases *in vivo*.

Cfd secreted by adipocytes was reported as a Pparγ downstream target gene [38]. We show the role of Cfd in stimulating Pparγ expression and adipocyte conversion. First, we identified Cfd as a gene regulated by Pparγ inducers. Second, agonistic regulation of Pparγ by GW7845 treatment in preadipocytes failed to acutely induce Cfd expression. Third, the selected pro-adipogenic small molecules did not exhibit Pparγ agonistic activities. Treatment with any of these compounds in combination with Pparγ agonist GW7845 for 24 hours additively increased the expression of aP2 but not that of Pparγ itself, further suggesting that GW7845 and these small molecules act through different mechanisms (Figure G in S1 File). Finally, ectopic overexpression and knockdown experiments showed that Cfd is sufficient and necessary for induction of Pparγ during adipocyte differentiation. Based on these, it is likely that Cfd can induce Pparγ expression, and in turn, Pparγ drives the expression of Cfd in adipocytes. However, currently we do not know the molecular mechanism by which Cfd regulates Pparγ expression in adipocytes. Similarly, the upstream factors regulated by the pro-adipogenic compounds (PT24 and PT26) inducing Cfd expression has not been investigated. In addition, it will be interesting to investigate the pro-adipogenic effects of these compounds in human adipocytes in the future.

The roles of Cfd have been well recognized in immune cells that protect against infection. In the immune system, hepatic C3b, by spontaneous hydrolysis of C3, associates with Cfd (adipsin) to cleave factor B, resulting in generation of C3 convertase [9,10]. Subsequently, C3a and C3b production activates the alternative pathway and protects against pathogen-induced infection. The role of complement system is also conserved in adipocyte biology. The C3a-C3aR signaling stimulates triglyceride synthesis in adipocytes [12]. Complement factors including Cfd, C3, C3aR, and Cfb are also produced by adipose tissues suggesting the dual effects in adipocytes and immune cells [9,10]. In line with this, our data show the action of secreted Cfd in promoting lipid accumulation and adipocyte differentiation by acting on C3aR, further indicating that the complement factors exert dual actions in adipocyte and immune cells. Although, we cannot totally exclude the possible roles of intracellular Cfd in adipogenesis, it seems clear that the immune cells and adipocytes in adipose tissues control glucose metabolism and insulin sensitivity. Thus, proper regulations on complement systems in adipose tissue may provide alternative strategies against metabolic diseases.

In conclusion, we identified Cfd as a gene induced by pro-adipogenic small molecules and further showed the roles of Cfd in activating C3aR, inducing Pparγ expression, and adipocyte differentiation. Our study provides evidence for the unidentified roles of Cfd in adipocytes and also highlights the utility of pro-adipogenic small molecules as tools to dissect adipocyte biology.

## Supporting Information

S1 File Supporting information. (PPTX)

## References

[1] Rosen CJ, Bouxsein ML. Mechanisms of disease: is osteoporosis the obesity of bone? Nat Clin Pract Rheumatol. 2006; 2: 35–43. 10.1038/ncprheum0070 16932650

[2] Bartelt A, Heeren J. Adipose tissue browning and metabolic health. Nat Rev Endocrinol. 2014; 10: 24–36. dio: 10.1038/nrendo.2013.204 24146030

[3] Waki H, Tontonoz P. Endocrine functions of adipose tissue. Annu Rev Patho. 2007; 2: 31–56 10.1146/annurev.pathol.2.010506.091859 . 18039092

[4] Tontonoz P, Nagy L, Alvarez JG, Thomazy VA, Evans RM. PPARgamma promotes monocyte/macrophage differentiation and uptake of oxidized LDL. Cell. 1998; 93: 241–252. 9568716 10.1016/s0092-8674(00)81575-5

[5] Tontonoz P, Spiegelman BM. Fat and beyond: the diverse biology of PPARgamma. Annu Rev Biochem. 2008; 77: 289–312. 10.1146/annurev.biochem.77.061307.091829 18518822

[6] Kim JH, Song J, Park KW. The multifaceted factor peroxisome proliferator-activated receptor gamma (PPARgamma) in metabolism, immunity, and cancer. Arch Pharm Res. 2015; 38: 302–312. 10.1007/s12272-015-0559-x 25579849

[7] Lefterova MI, Zhang Y, Steger DJ, Schupp M, Schug J, Cristancho A, et al PPARgamma and C/EBP factors orchestrate adipocyte biology via adjacent binding on a genome-wide scale. Genes Dev. 2008; 22: 2941–2952. 10.1101/gad.1709008 18981473 PMC2577797

[8] Morgan BP, Harris CL. Complement, a target for therapy in inflammatory and degenerative diseases. Nat Rev Drug Discov. 2015; 14: 857–877. 10.1038/nrd4657 26493766 PMC7098197

[9] Zipfel PF, Skerka C. Complement regulators and inhibitory proteins. Nat Rev Immunol. 2009; 9: 729–740. 10.1038/nri2620 19730437

[10] Pattrick M, Luckett J, Yue L, Stover C. Dual role of complement in adipose tissue. Mol Immunol. 2009; 46: 755–760. 10.1016/j.molimm.2008.09.013 18954909

[11] Baldo A, Sniderman AD, St-Luce S, Avramoglu RK, Maslowska M, Hoang B, et al The adipsin-acylation stimulating protein system and regulation of intracellular triglyceride synthesis. J Clin Invest. 1993; 92: 1543–1547. 10.1172/JCI116733 8376604 PMC288301

[12] Mamane Y, Chung Chan C, Lavallee G, Morin N, Xu LJ, Huang J, et al The C3a anaphylatoxin receptor is a key mediator of insulin resistance and functions by modulating adipose tissue macrophage infiltration and activation. Diabetes. 2009; 58: 2006–2017. 10.2337/db09-0323 19581423 PMC2731537

[13] Engstrom G, Hedblad B, Eriksson KF, Janzon L, Lindgarde F. Complement C3 is a risk factor for the development of diabetes: a population-based cohort study. Diabetes. 2005; 54: 570–575. 15677517 10.2337/diabetes.54.2.570

[14] Xia Z, Sniderman AD, Cianflone K. Acylation-stimulating protein (ASP) deficiency induces obesity resistance and increased energy expenditure in ob/ob mice. J Biol Chem. 2002; 277: 45874–45879. 10.1074/jbc.M207281200 12244109

[15] Paglialunga S, Schrauwen P, Roy C, Moonen-Kornips E, Lu H, Hesselink MK, et al Reduced adipose tissue triglyceride synthesis and increased muscle fatty acid oxidation in C5L2 knockout mice. J Endocrinol. 2007; 194: 293–304. 10.1677/JOE-07-0205 17641279

[16] Waki H, Park KW, Mitro N, Pei L, Damoiseaux R, Wilpitz DC, et al The small molecule harmine is an antidiabetic cell-type-specific regulator of PPARgamma expression. Cell Metab. 2007; 5: 357–370. 10.1016/j.cmet.2007.03.010 17488638

[17] Park KW, Waki H, Kim WK, Davies BS, Young SG, Parhami F, et al The small molecule phenamil induces osteoblast differentiation and mineralization. Mol Cell Biol. 2009; 29: 3905–3914. 10.1128/MCB.00002-09 19433444 PMC2704753

[18] Park KW, Waki H, Choi SP, Park KM, Tontonoz P. The small molecule phenamil is a modulator of adipocyte differentiation and PPARgamma expression. J Lipid Res. 2010; 51: 2775–2784. 10.1194/jlr.M008490 20519739 PMC2918460

[19] Choy LN, Rosen BS, Spiegelman BM. Adipsin and an endogenous pathway of complement from adipose cells. J Biol Chem. 1992; 267: 12736–12741. 1618777

[20] Baek K, Park HJ, Hwang HR, Baek JH. Propranolol attenuates calorie restriction- and high calorie diet-induced bone marrow adiposity. BMB Rep. 2014; 47: 587–592. 25248563 10.5483/BMBRep.2014.47.10.176 PMC4261518

[21] Song NJ, Yoon HJ, Kim KH, Jung SR, Jang WS, Seo CR, et al Butein is a novel anti-adipogenic compound. J Lipid Res. 2013; 54: 1385–1396. 10.1194/jlr.M035576 23468131 PMC3622332

[22] Park KW, Waki H, Villanueva CJ, Monticelli LA, Hong C, Kang S, et al Inhibitor of DNA binding 2 is a small molecule-inducible modulator of peroxisome proliferator-activated receptor-gamma expression and adipocyte differentiation. Mol Endocrinol. 2008; 22: 2038–2048. 10.1210/me 18562627 PMC2631374

[23] Kang S, Bennett CN, Gerin I, Rapp LA, Hankenson KD, MacDougald OA. Wnt signaling stimulates osteoblastogenesis of mesenchymal precursors by suppressing CCAAT/enhancer-binding protein alpha and peroxisome proliferator-activated receptor gamma. J Biol Chem. 2007; 282: 14515–14524. 17351296 10.1074/jbc.M700030200

[24] Kim CS, Kawada T, Yoo H, Kwon BS, Yu R. Macrophage inflammatory protein-related protein-2, a novel CC chemokine, can regulate preadipocyte migration and adipocyte differentiation. FEBS Lett. 2003; 548: 125–130. 12885419 10.1016/s0014-5793(03)00728-2

[25] White RT, Damm D, Hancock N, Rosen BS, Lowell BB, Usher P, et al Human adipsin is identical to complement factor D and is expressed at high levels in adipose tissue. J Biol Chem. 1992 267: 9210–9213. 1374388

[26] Yasruel Z, Cianflone K, Sniderman AD, Rosenbloom M, Walsh M, Rodriguez MA. Effect of acylation stimulating protein on the triacylglycerol synthetic pathway of human adipose tissue. Lipids. 1991; 26: 495–499. 1943492 10.1007/BF02536592

[27] Ding S, Schultz PG. A role for chemistry in stem cell biology. Nat Biotechnol. 2004; 22: 833–840. 10.1038/nbt987 15229546

[28] Lassar AB, Paterson BM, Weintraub H. Transfection of a DNA locus that mediates the conversion of 10T1/2 fibroblasts to myoblasts. Cell. 1986; 47: 649–656. 2430720 2430720 10.1016/0092-8674(86)90507-6

[29] Lehmann JM, Moore LB, Smith-Oliver TA, Wilkison WO, Willson TM, Kliewer SA. An antidiabetic thiazolidinedione is a high affinity ligand for peroxisome proliferator-activated receptor gamma (PPAR gamma). J Biol Chem. 1995; 270: 12953–12956. 7768881 10.1074/jbc.270.22.12953

[30] Tapscott SJ, Davis RL, Thayer MJ, Cheng PF, Weintraub H, Lassar AB. MyoD1: a nuclear phosphoprotein requiring a Myc homology region to convert fibroblasts to myoblasts. Science. 1988; 242: 405–411. 3175662 10.1126/science.3175662

[31] Choi JH, Banks AS, Estall JL, Kajimura S, Bostrom P, Laznik D, et al Anti-diabetic drugs inhibit obesity-linked phosphorylation of PPARgamma by Cdk5. Nature. 2010; 466: 451–456. 10.1038/nature09291 20651683 PMC2987584

[32] Choi JH, Banks AS, Kamenecka TM, Busby SA, Chalmers MJ, Kumar N, et al Antidiabetic actions of a non-agonist PPARgamma ligand blocking Cdk5-mediated phosphorylation. Nature. 2011; 477: 477–481. 10.1038/nature10383 21892191 PMC3179551

[33] Flier JS, Cook KS, Usher P, Spiegelman BM. Severely impaired adipsin expression in genetic and acquired obesity. Science. 1987; 237: 405–408. 3299706 10.1126/science.3299706

[34] Lowell BB, Napolitano A, Usher P, Dulloo AG, Rosen BS, Spiegelman BM, et al Reduced adipsin expression in murine obesity: effect of age and treatment with the sympathomimetic-thermogenic drug mixture ephedrine and caffeine. Endocrinology. 1990; 126: 1514–1520. 10.1210/endo-126-3-1514 2307116

[35] Rosen BS, Cook KS, Yaglom J, Groves DL, Volanakis JE, Damm D, et al Adipsin and complement factor D: an immune-related defect in obesity. Science. 1989; 244 (4911): 1483–1487. 2734615 10.1126/science.2734615

[36] Lo JC, Ljubicic S, Leibiger B, Kern M, Leibiger IB, Moede T, et al Adipsin is an adipokine that improves β cell function in diabetes. Cell. 2014; 158 (1): 41–53. 10.1016/j.cell.2014.06.005 24995977 PMC4128197

[37] Sissons JG, West RJ, Fallows J, Williams DG, Boucher BJ, Amos N, et al The complement abnormalities of lipodystrophy. N Engl J Med. 1976; 294: 461–465. 10.1056/NEJM197602262940902 1246331

[38] Brown JD, Plutzky J. Peroxisome proliferator-activated receptors as transcriptional nodal points and therapeutic targets. Circulation. 2007; 115: 518–533. 17261671 10.1161/CIRCULATIONAHA.104.475673

