# Supplementary material for: Small Molecule-Induced Complement Factor D (Adipsin) Promotes Lipid Accumulation and Adipocyte Differentiation
Source: PLoS One. 2016 Sep 9;11(9):e0162228. doi: 10.1371/journal.pone.0162228 (PMC5017651; doi:10.1371/journal.pone.0162228)
Supplement: S1 File — (PPTX) [file pone.0162228.s001.pptx]

## Slide 1
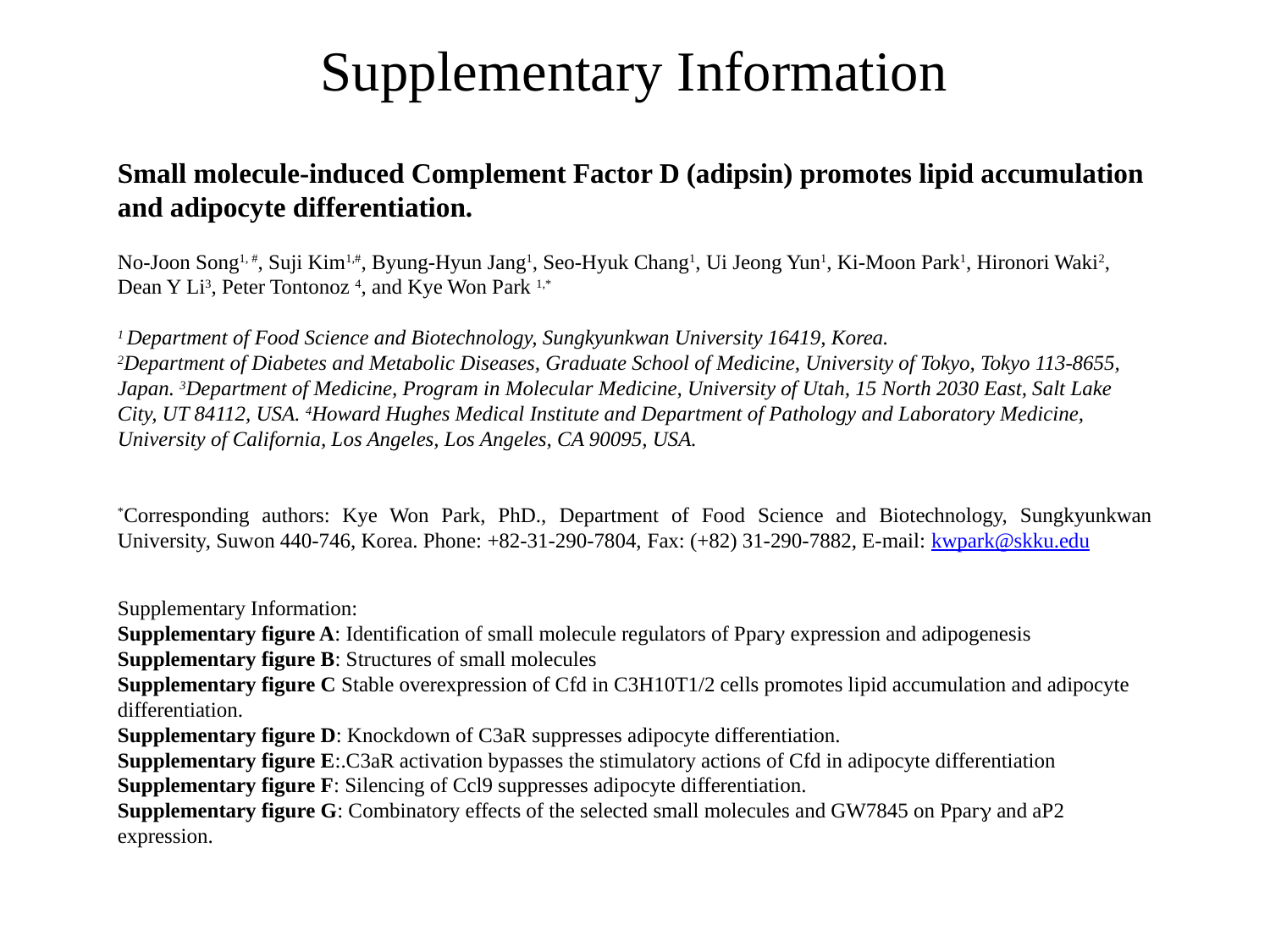

# Supplementary Information
Small molecule-induced Complement Factor D (adipsin) promotes lipid accumulation and adipocyte differentiation.
No-Joon Song1, #, Suji Kim1,#, Byung-Hyun Jang1, Seo-Hyuk Chang1, Ui Jeong Yun1, Ki-Moon Park1, Hironori Waki2, Dean Y Li3, Peter Tontonoz 4, and Kye Won Park 1,*
1 Department of Food Science and Biotechnology, Sungkyunkwan University 16419, Korea.
2Department of Diabetes and Metabolic Diseases, Graduate School of Medicine, University of Tokyo, Tokyo 113-8655, Japan. 3Department of Medicine, Program in Molecular Medicine, University of Utah, 15 North 2030 East, Salt Lake City, UT 84112, USA. 4Howard Hughes Medical Institute and Department of Pathology and Laboratory Medicine, University of California, Los Angeles, Los Angeles, CA 90095, USA.
*Corresponding authors: Kye Won Park, PhD., Department of Food Science and Biotechnology, Sungkyunkwan University, Suwon 440-746, Korea. Phone: +82-31-290-7804, Fax: (+82) 31-290-7882, E-mail: kwpark@skku.edu
Supplementary Information:
Supplementary figure A: Identification of small molecule regulators of Pparγ expression and adipogenesis
Supplementary figure B: Structures of small molecules
Supplementary figure C Stable overexpression of Cfd in C3H10T1/2 cells promotes lipid accumulation and adipocyte differentiation.
Supplementary figure D: Knockdown of C3aR suppresses adipocyte differentiation.
Supplementary figure E:.C3aR activation bypasses the stimulatory actions of Cfd in adipocyte differentiation
Supplementary figure F: Silencing of Ccl9 suppresses adipocyte differentiation.
Supplementary figure G: Combinatory effects of the selected small molecules and GW7845 on Pparγ and aP2 expression.

## Slide 2
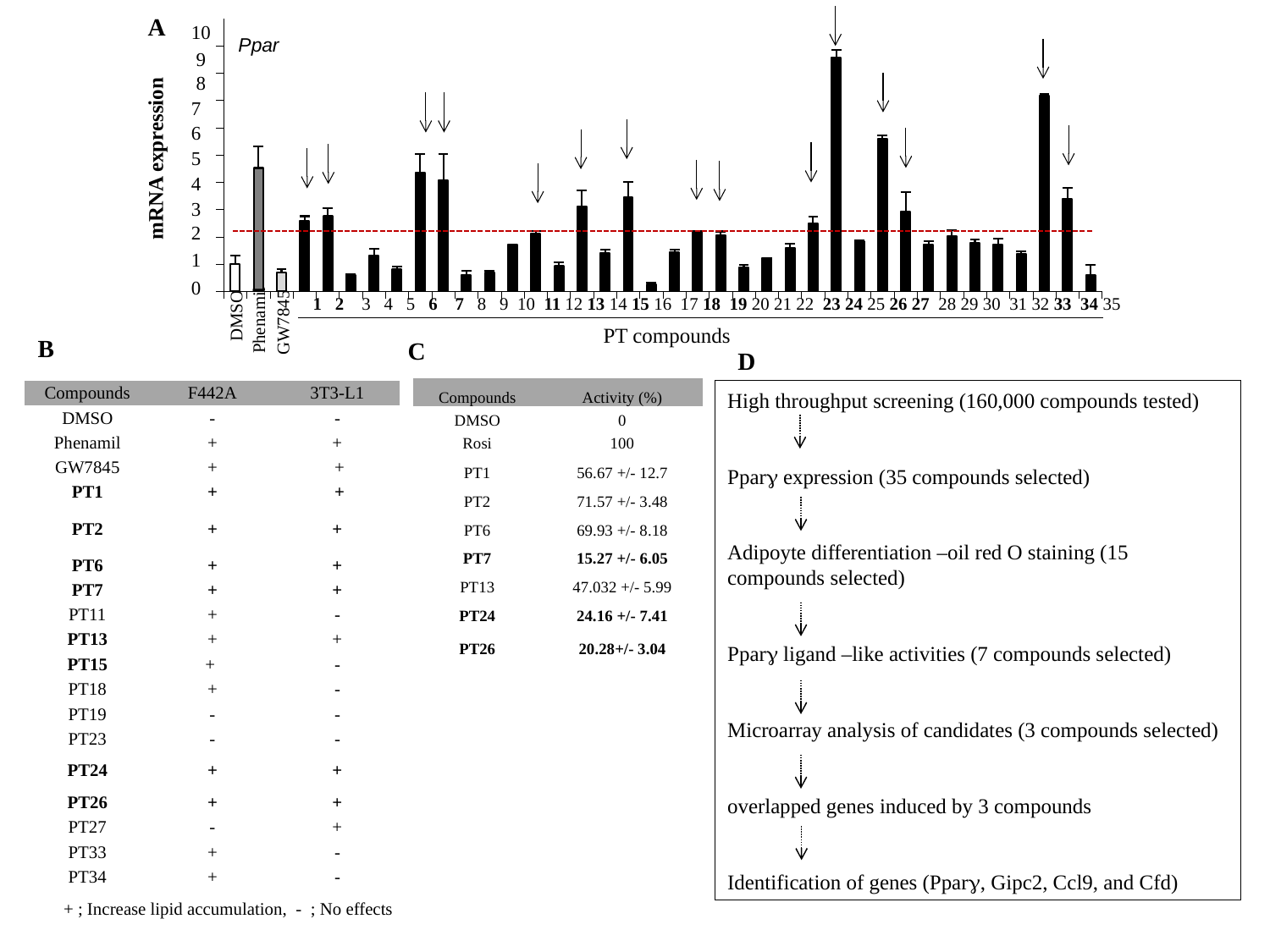

### Chart: Pparγ
| Category | |
|---|---|
| DMSO | 0.35934863870087475 |
| PHENMAIL | 1.626595420732066 |
| GW | 0.2504131830741316 |
| PT1 | 0.9284179632943106 |
| PT2 | 0.9907812535610167 |
| PT3 | 0.21965462286331822 |
| PT4 | 0.4743268549854486 |
| PT5 | 0.29430129377759023 |
| PT6 | 1.562992045360863 |
| PT7 | 1.4654872148029519 |
| PT8 | 0.219951675469668 |
| PT9 | 0.24815022717352792 |
| PT10 | 0.6152974684928969 |
| PT11 | 0.7572327544116045 |
| PT12 | 0.33209773598021575 |
| PT13 | 1.1137632457584814 |
| PT14 | 0.5000794024424172 |
| PT15 | 1.242063909399208 |
| PT16 | 0.08941961797811043 |
| PT17 | 0.5125241301737177 |
| PT18 | 0.7863871861760604 |
| PT19 | 0.7440501928282947 |
| PT20 | 0.31545629549794896 |
| PT21 | 0.44073992218316543 |
| PT22 | 0.5701582260099995 |
| PT23 | 0.8996267849350347 |
| PT24 | 3.081611521484856 |
| PT25 | 0.6576744811266216 |
| PT26 | 2.012507480262977 |
| PT27 | 1.0559523218507527 |
| PT28 | 0.6184224528151137 |
| PT29 | 0.7273518879848865 |
| PT30 | 0.641753014238839 |
| PT31 | 0.6179130956396077 |
| PT32 | 0.4926822038401439 |
| PT33 | 2.5765801581846715 |
| PT34 | 1.221293458893183 |
| PT36 | 0.2184991910629284 |A
10
 9
 8
7
6
5
4
3
2
1
0
mRNA expression
 1 2 3 4 5 6 7 8 9 10 11 12 13 14 15 16 17 18 19 20 21 22 23 24 25 26 27 28 29 30 31 32 33 34 35
DMSO
Phenamil
GW7845
PT compounds
B
C
D
| Compounds | Activity (%) |
| --- | --- |
| DMSO | 0 |
| Rosi | 100 |
| PT1 | 56.67 +/- 12.7 |
| PT2 | 71.57 +/- 3.48 |
| PT6 | 69.93 +/- 8.18 |
| PT7 | 15.27 +/- 6.05 |
| PT13 | 47.032 +/- 5.99 |
| PT24 | 24.16 +/- 7.41 |
| PT26 | 20.28+/- 3.04 |
High throughput screening (160,000 compounds tested)
Pparγ expression (35 compounds selected)
Adipoyte differentiation –oil red O staining (15 compounds selected)
Pparγ ligand –like activities (7 compounds selected)
Microarray analysis of candidates (3 compounds selected)
overlapped genes induced by 3 compounds
Identification of genes (Pparγ, Gipc2, Ccl9, and Cfd)
| Compounds | F442A | 3T3-L1 |
| --- | --- | --- |
| DMSO | - | - |
| Phenamil | + | + |
| GW7845 | + | + |
| PT1 | + | + |
| PT2 | + | + |
| PT6 | + | + |
| PT7 | + | + |
| PT11 | + | - |
| PT13 | + | + |
| PT15 | + | - |
| PT18 | + | - |
| PT19 | - | - |
| PT23 | - | - |
| PT24 | + | + |
| PT26 | + | + |
| PT27 | - | + |
| PT33 | + | - |
| PT34 | + | - |
+ ; Increase lipid accumulation, - ; No effects

## Slide 3
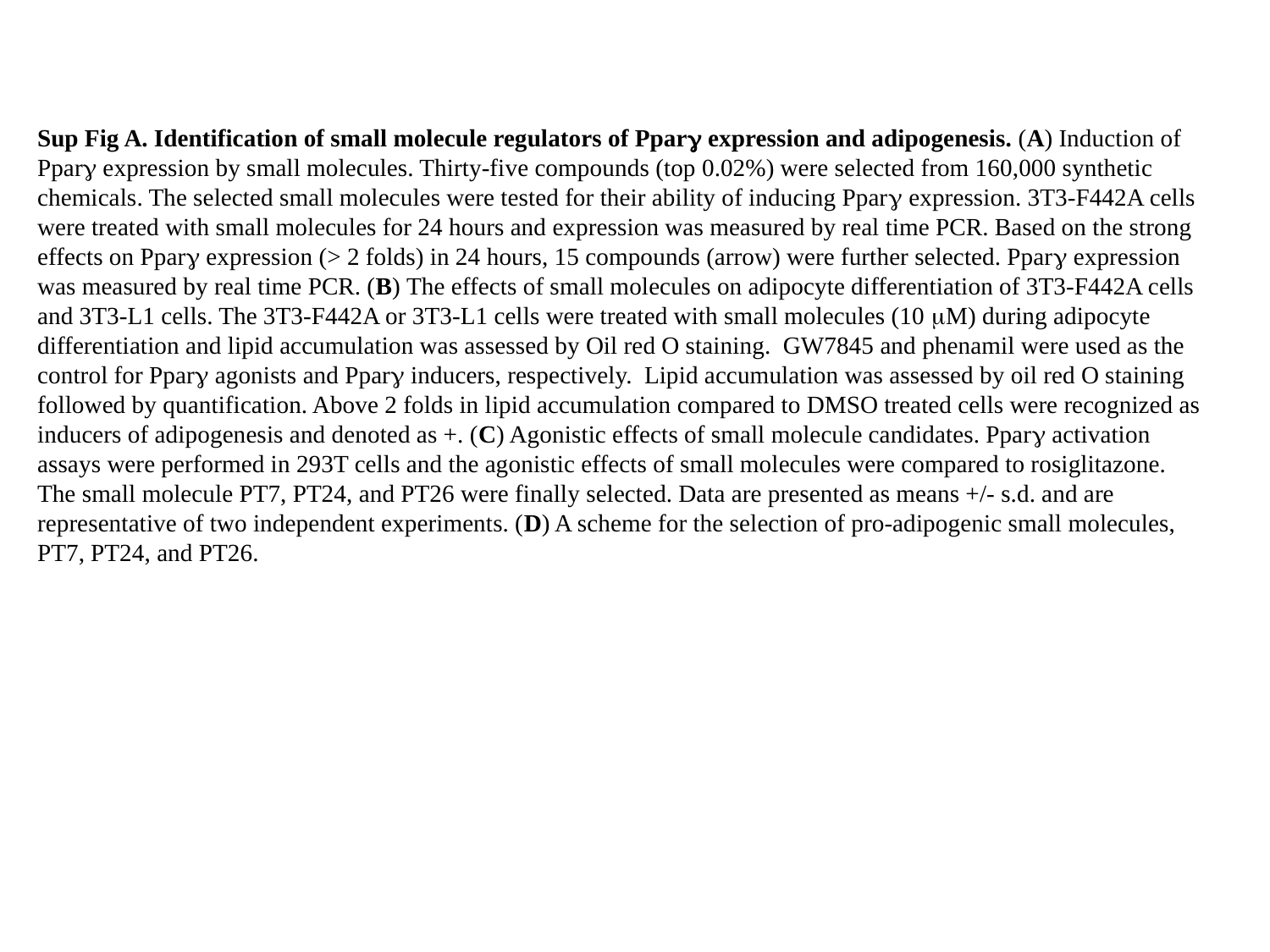

Sup Fig A. Identification of small molecule regulators of Pparγ expression and adipogenesis. (A) Induction of Pparγ expression by small molecules. Thirty-five compounds (top 0.02%) were selected from 160,000 synthetic chemicals. The selected small molecules were tested for their ability of inducing Pparγ expression. 3T3-F442A cells were treated with small molecules for 24 hours and expression was measured by real time PCR. Based on the strong effects on Pparγ expression (> 2 folds) in 24 hours, 15 compounds (arrow) were further selected. Pparγ expression was measured by real time PCR. (B) The effects of small molecules on adipocyte differentiation of 3T3-F442A cells and 3T3-L1 cells. The 3T3-F442A or 3T3-L1 cells were treated with small molecules (10 μM) during adipocyte differentiation and lipid accumulation was assessed by Oil red O staining. GW7845 and phenamil were used as the control for Pparγ agonists and Pparγ inducers, respectively. Lipid accumulation was assessed by oil red O staining followed by quantification. Above 2 folds in lipid accumulation compared to DMSO treated cells were recognized as inducers of adipogenesis and denoted as +. (C) Agonistic effects of small molecule candidates. Pparγ activation assays were performed in 293T cells and the agonistic effects of small molecules were compared to rosiglitazone. The small molecule PT7, PT24, and PT26 were finally selected. Data are presented as means +/- s.d. and are representative of two independent experiments. (D) A scheme for the selection of pro-adipogenic small molecules, PT7, PT24, and PT26.

## Slide 4
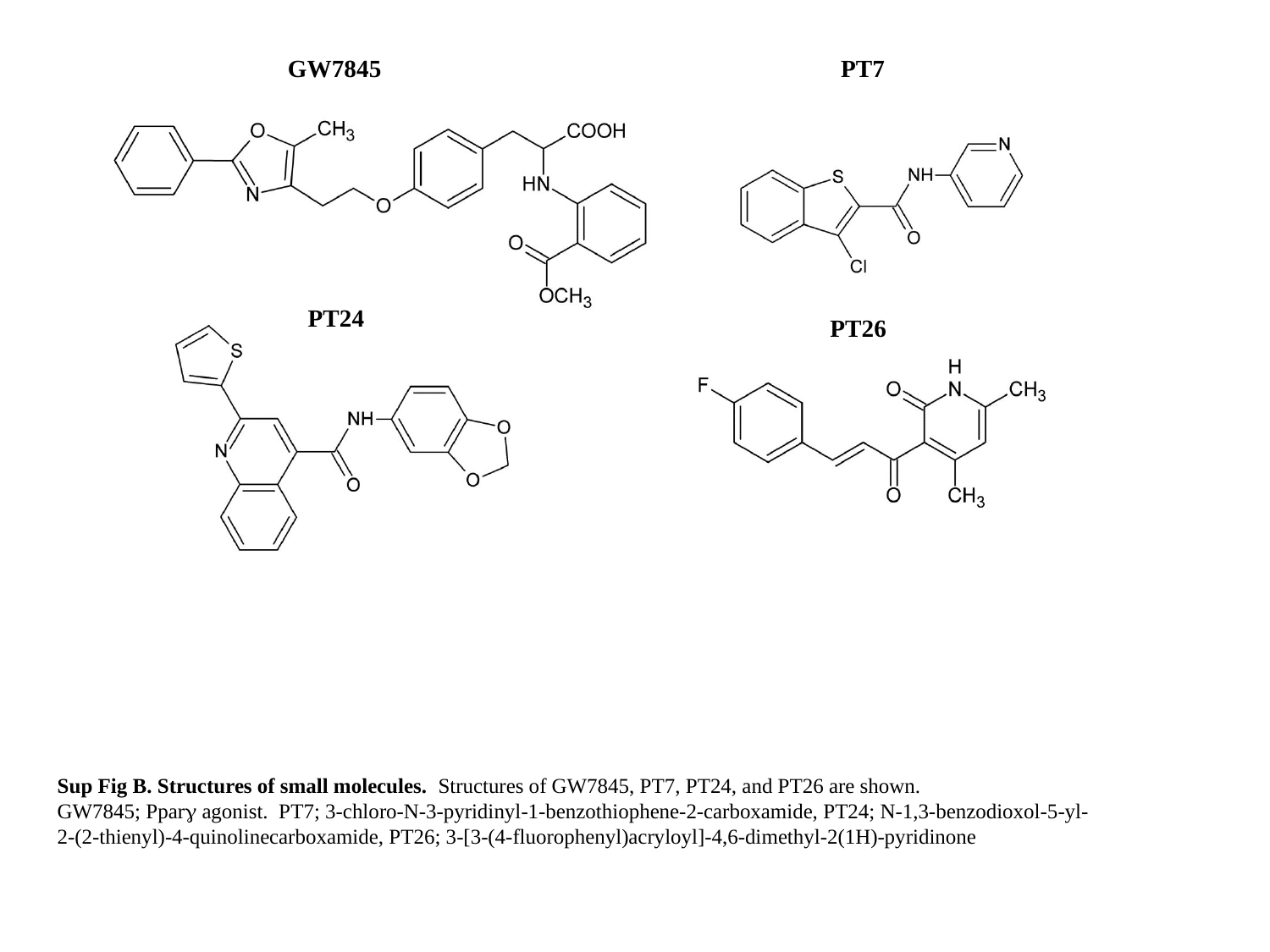

GW7845
PT7
PT24
PT26
Sup Fig B. Structures of small molecules. Structures of GW7845, PT7, PT24, and PT26 are shown.
GW7845; Pparγ agonist. PT7; 3-chloro-N-3-pyridinyl-1-benzothiophene-2-carboxamide, PT24; N-1,3-benzodioxol-5-yl-2-(2-thienyl)-4-quinolinecarboxamide, PT26; 3-[3-(4-fluorophenyl)acryloyl]-4,6-dimethyl-2(1H)-pyridinone

## Slide 5
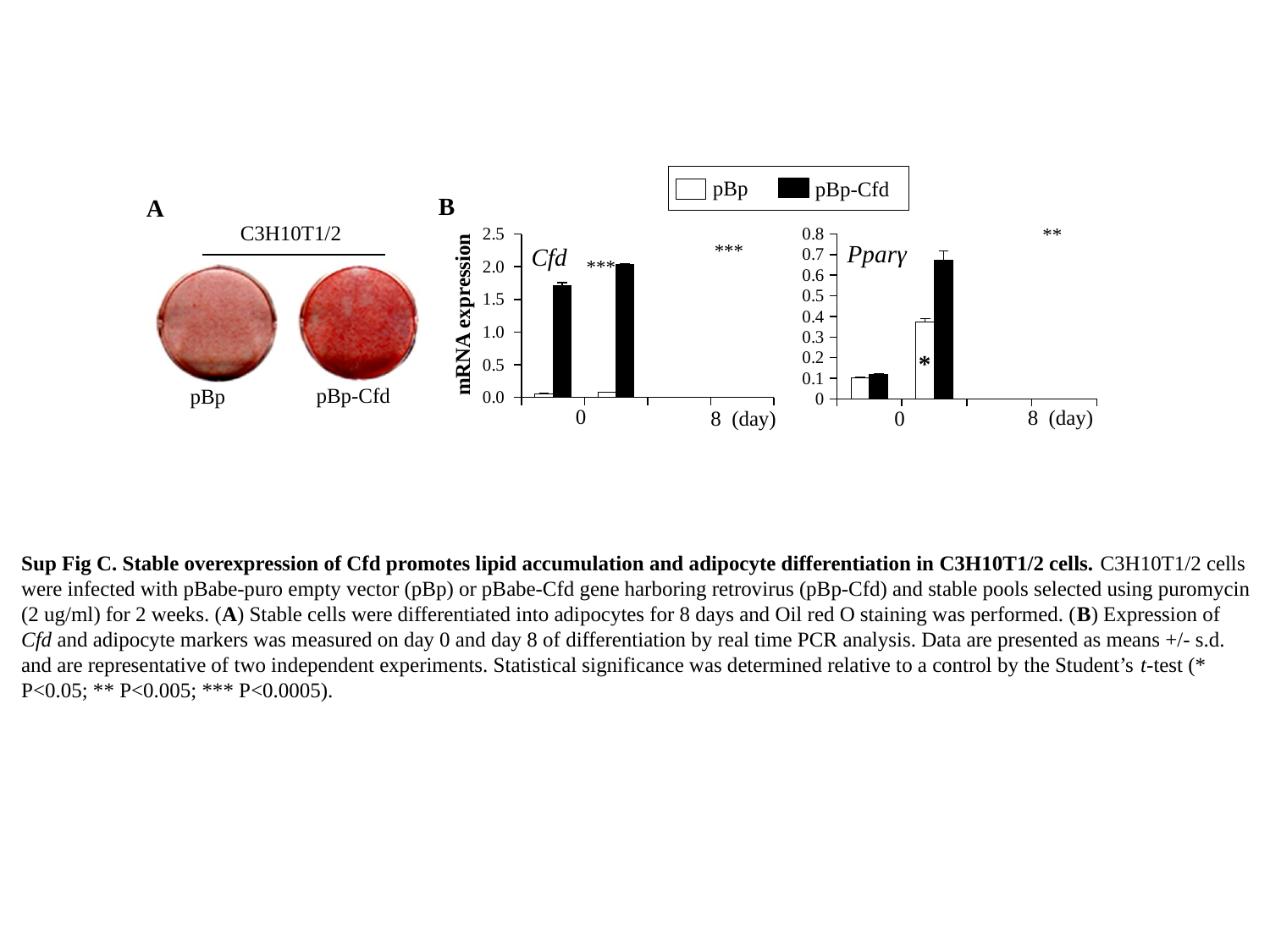

pBp
pBp-Cfd
B
A
C3H10T1/2
pBp-Cfd
pBp
[unsupported chart]
Pparγ
[unsupported chart]
Cfd
**
***
***
mRNA expression
*
0
8 (day)
8 (day)
0
Sup Fig C. Stable overexpression of Cfd promotes lipid accumulation and adipocyte differentiation in C3H10T1/2 cells. C3H10T1/2 cells were infected with pBabe-puro empty vector (pBp) or pBabe-Cfd gene harboring retrovirus (pBp-Cfd) and stable pools selected using puromycin (2 ug/ml) for 2 weeks. (A) Stable cells were differentiated into adipocytes for 8 days and Oil red O staining was performed. (B) Expression of Cfd and adipocyte markers was measured on day 0 and day 8 of differentiation by real time PCR analysis. Data are presented as means +/- s.d. and are representative of two independent experiments. Statistical significance was determined relative to a control by the Student’s t-test (* P<0.05; ** P<0.005; *** P<0.0005).

## Slide 6
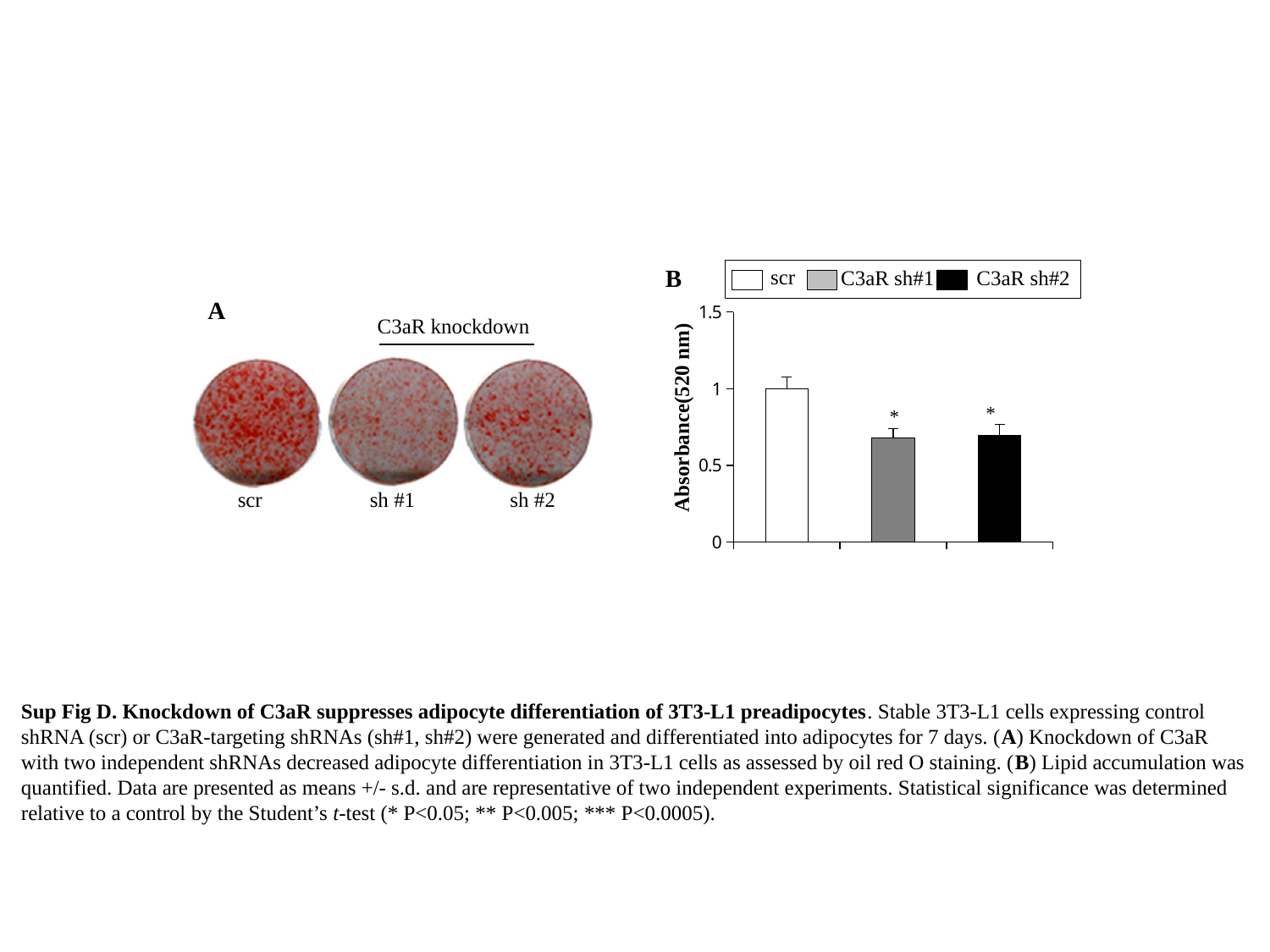

B
scr
C3aR sh#1 C3aR sh#2
A
### Chart
| Category | |
|---|---|Absorbance(520 nm)
C3aR knockdown
sh #1
sh #2
scr
*
*
Sup Fig D. Knockdown of C3aR suppresses adipocyte differentiation of 3T3-L1 preadipocytes. Stable 3T3-L1 cells expressing control shRNA (scr) or C3aR-targeting shRNAs (sh#1, sh#2) were generated and differentiated into adipocytes for 7 days. (A) Knockdown of C3aR with two independent shRNAs decreased adipocyte differentiation in 3T3-L1 cells as assessed by oil red O staining. (B) Lipid accumulation was quantified. Data are presented as means +/- s.d. and are representative of two independent experiments. Statistical significance was determined relative to a control by the Student’s t-test (* P<0.05; ** P<0.005; *** P<0.0005).

## Slide 7
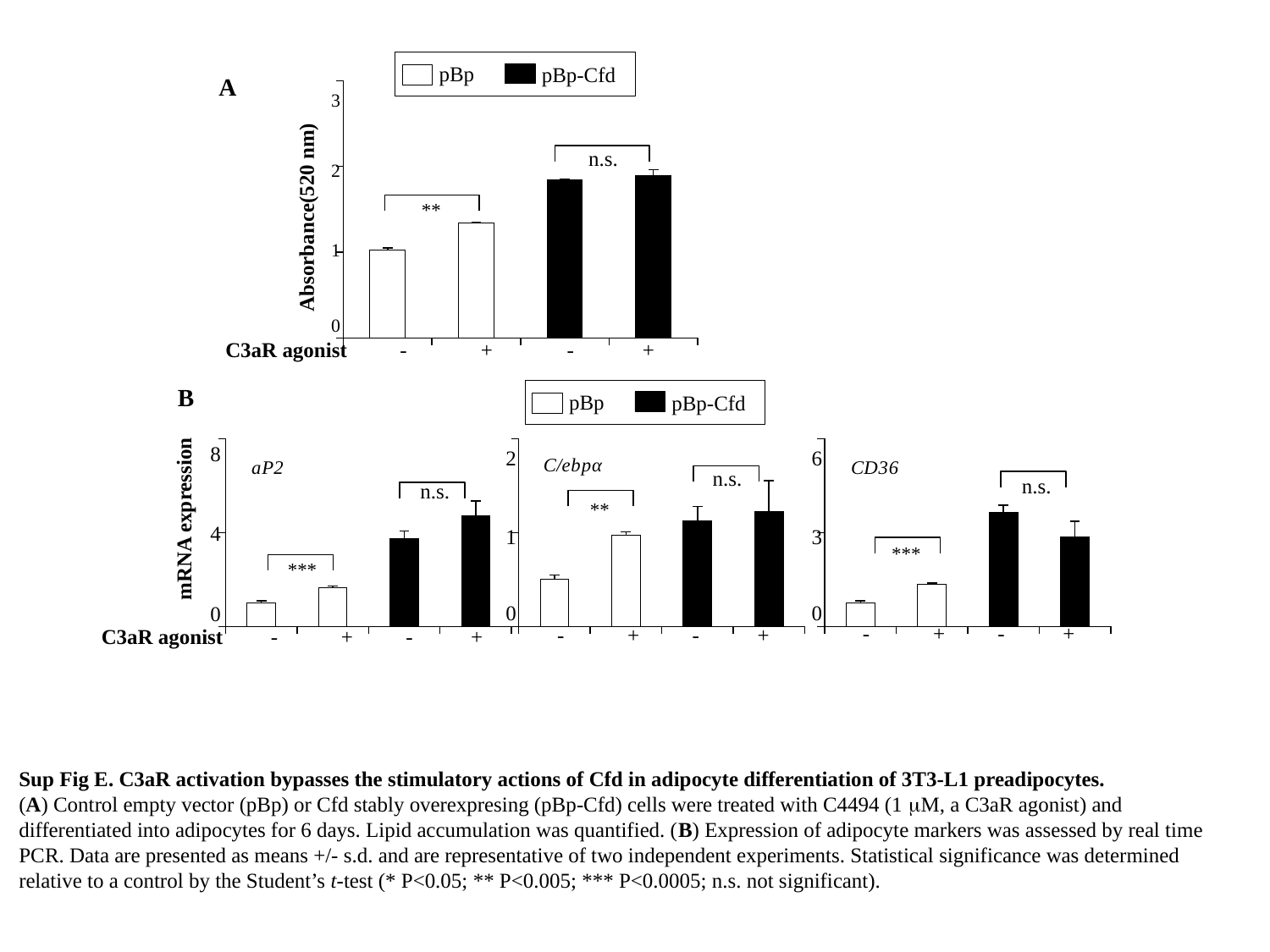

pBp
pBp-Cfd
A
### Chart
| Category | |
|---|---|
| 0 | 1.0249999999999941 |
| 1 | 1.33875 |
| 0 | 1.8399999999999932 |
| 1 | 1.88875 |3
2
1
0
n.s.
**
Absorbance(520 nm)
C3aR agonist - + - +
B
pBp
pBp-Cfd
8
4
0
### Chart
| Category | |
|---|---|
| 0 | 1.0 |
| 1 | 1.654287166745218 |
| 0 | 3.757290913530384 |
| 1 | 4.699454680692435 |
### Chart
| Category | |
|---|---|
| 0 | 1.0 |
| 1 | 1.9473641560363415 |
| 0 | 2.242942579815422 |
| 1 | 2.4554471528845543 |
### Chart
| Category | |
|---|---|
| 0 | 1.0 |
| 1 | 1.7841034347475093 |
| 0 | 4.8566628671974685 |
| 1 | 3.827742012218857 |2
1
0
6
3
0
n.s.
n.s.
n.s.
**
mRNA expression
***
***
- + - +
- + - +
C3aR agonist - + - +
Sup Fig E. C3aR activation bypasses the stimulatory actions of Cfd in adipocyte differentiation of 3T3-L1 preadipocytes.
(A) Control empty vector (pBp) or Cfd stably overexpresing (pBp-Cfd) cells were treated with C4494 (1 μM, a C3aR agonist) and differentiated into adipocytes for 6 days. Lipid accumulation was quantified. (B) Expression of adipocyte markers was assessed by real time PCR. Data are presented as means +/- s.d. and are representative of two independent experiments. Statistical significance was determined relative to a control by the Student’s t-test (* P<0.05; ** P<0.005; *** P<0.0005; n.s. not significant).

## Slide 8
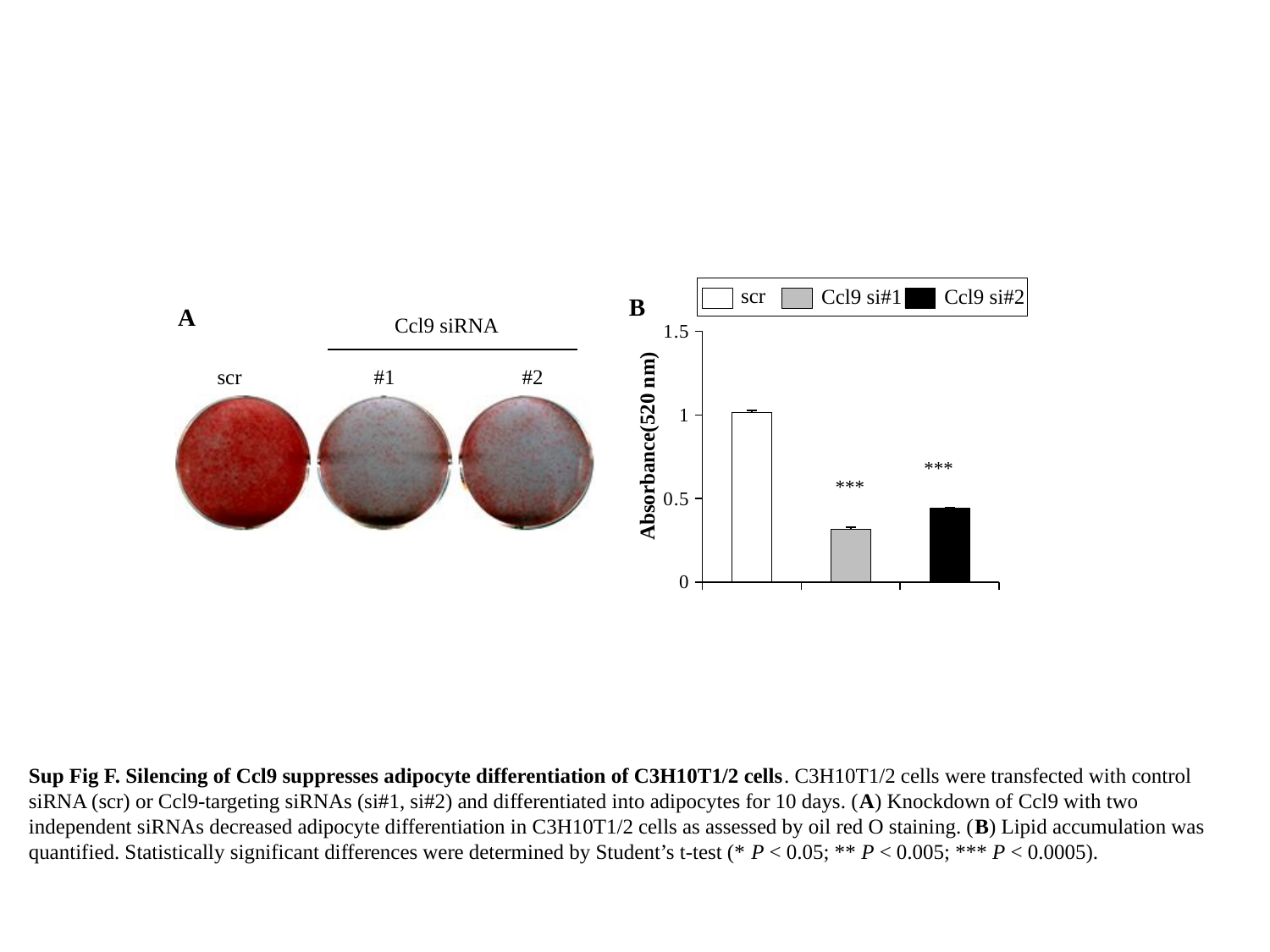

scr
Ccl9 si#1 Ccl9 si#2
B
A
 Ccl9 siRNA
### Chart
| Category | |
|---|---|
| Scr | 1.01413881748072 |
| #1 | 0.3168380462724947 |
| #2 | 0.44280205655526983 |scr #1 #2
Absorbance(520 nm)
***
***
Sup Fig F. Silencing of Ccl9 suppresses adipocyte differentiation of C3H10T1/2 cells. C3H10T1/2 cells were transfected with control siRNA (scr) or Ccl9-targeting siRNAs (si#1, si#2) and differentiated into adipocytes for 10 days. (A) Knockdown of Ccl9 with two independent siRNAs decreased adipocyte differentiation in C3H10T1/2 cells as assessed by oil red O staining. (B) Lipid accumulation was quantified. Statistically significant differences were determined by Student’s t-test (* P < 0.05; ** P < 0.005; *** P < 0.0005).

## Slide 9
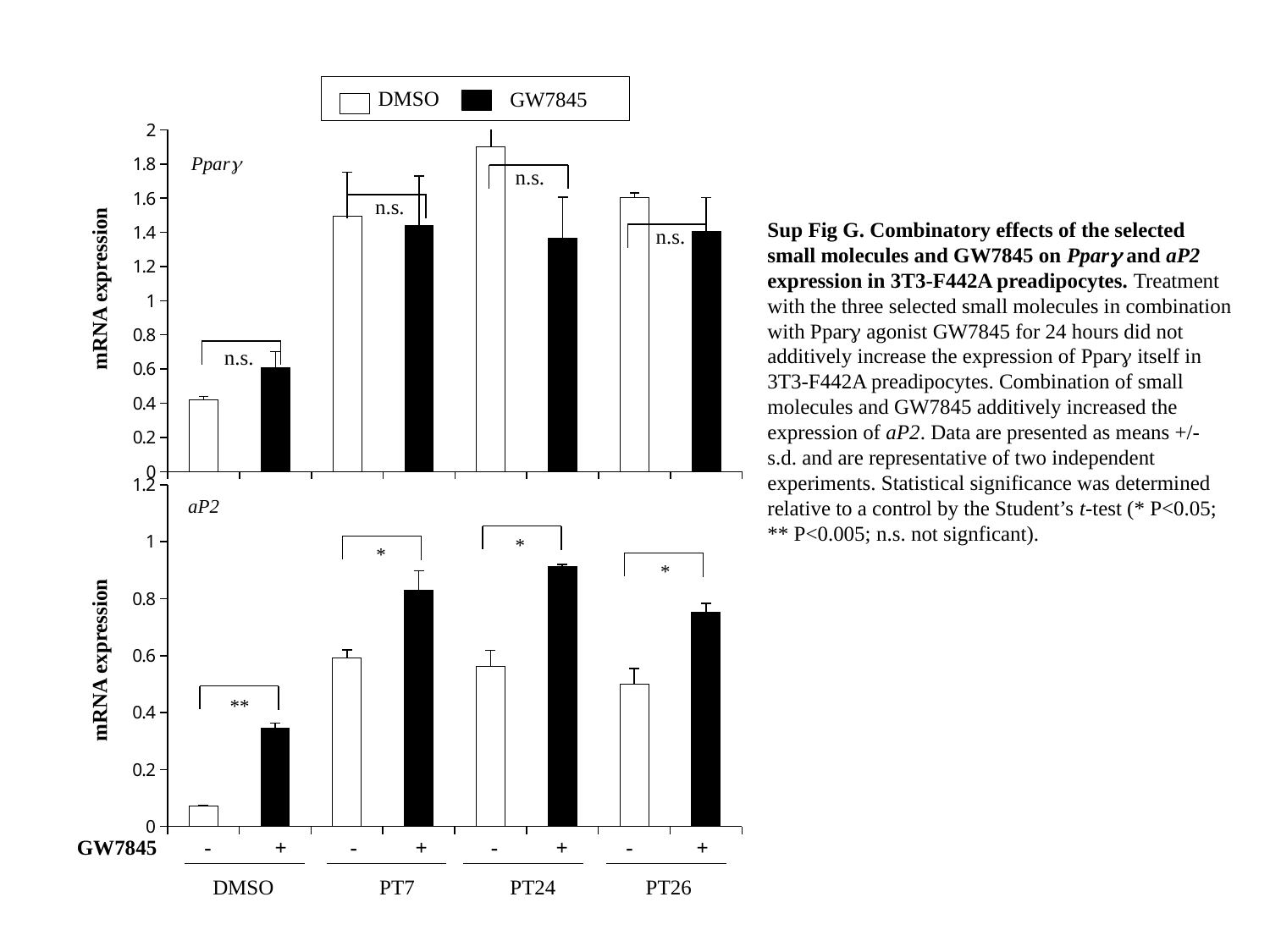

DMSO
GW7845
### Chart
| Category | |
|---|---|Pparγ
n.s.
n.s.
Sup Fig G. Combinatory effects of the selected small molecules and GW7845 on Pparγ and aP2 expression in 3T3-F442A preadipocytes. Treatment with the three selected small molecules in combination with Pparγ agonist GW7845 for 24 hours did not additively increase the expression of Pparγ itself in 3T3-F442A preadipocytes. Combination of small molecules and GW7845 additively increased the expression of aP2. Data are presented as means +/- s.d. and are representative of two independent experiments. Statistical significance was determined relative to a control by the Student’s t-test (* P<0.05; ** P<0.005; n.s. not signficant).
n.s.
mRNA expression
n.s.
### Chart
| Category | |
|---|---|aP2
*
*
*
mRNA expression
**
GW7845 - + - + - + - +
DMSO PT7 PT24 PT26
